# Adaptive Sharding for UAV Networks: A Deep Reinforcement Learning Approach to Blockchain Optimization

**DOI:** 10.3390/s24227279

**Published:** 2024-11-14

**Authors:** Kaiyin Lu, Xinguang Zhang, Tianbo Zhai, Mengjie Zhou

**Affiliations:** 1 Department of Computer Science, School of Information Science and Technology, Jinan University, Guangzhou 510632, Chinaztb0016@stu2022.jnu.edu.cn (T.Z.); 2 The Erik Jonsson School of Engineering and Computer Science, The University of Texas at Dallas, Richardson, TX 75080, USA; xinguang.zhang@ieee.org; 3 Department of Computer Science, University of Bristol, Bristol BS8 1QU, UK

**Keywords:** UAV network, blockchain technology, adaptive sharding, A3C algorithm

## Abstract

As unmanned aerial vehicle (UAV) technology expands into diverse applications, the demand for enhanced performance intensifies. Blockchain sharding technology offers promising avenues for improving data processing capabilities and security in drone networks. However, the inherent mobility of UAVs and their dynamic operational environment pose significant challenges to conventional sharding techniques, often resulting in communication latencies and data synchronization delays that compromise efficiency. This study presents a novel blockchain-based adaptive sharding framework specifically designed for UAV ecosystems. Our research extends beyond improving data transmission rates to encompass an enhanced Asynchronous Advantage Actor–Critic algorithm, tailored to address long-term optimization objectives in aerial networks. The proposed optimizations focus on dual objectives: enhancing data security while concurrently accelerating processing speeds. By addressing the limitations of traditional approaches, this work aims to facilitate seamless communication and foster innovation in UAV networks. The adaptive sharding framework, coupled with the refined A3C algorithm, presents a comprehensive solution to the unique challenges faced by mobile aerial systems in blockchain implementation.

## 1. Introduction

Unmanned aerial vehicle (UAV) network systems represent a sophisticated, self-organizing mesh of airborne units that utilize wireless connectivity for data transmission, task coordination, and location synchronization. These systems demonstrate remarkable versatility, finding applications in diverse domains ranging from military surveillance to disaster response and precision agriculture. The efficiency and seamless cooperation within UAV networks are facilitated by specialized routing protocols and optimized network topologies, thereby revolutionizing aerial operations across multiple industries.

**Figure 1:**
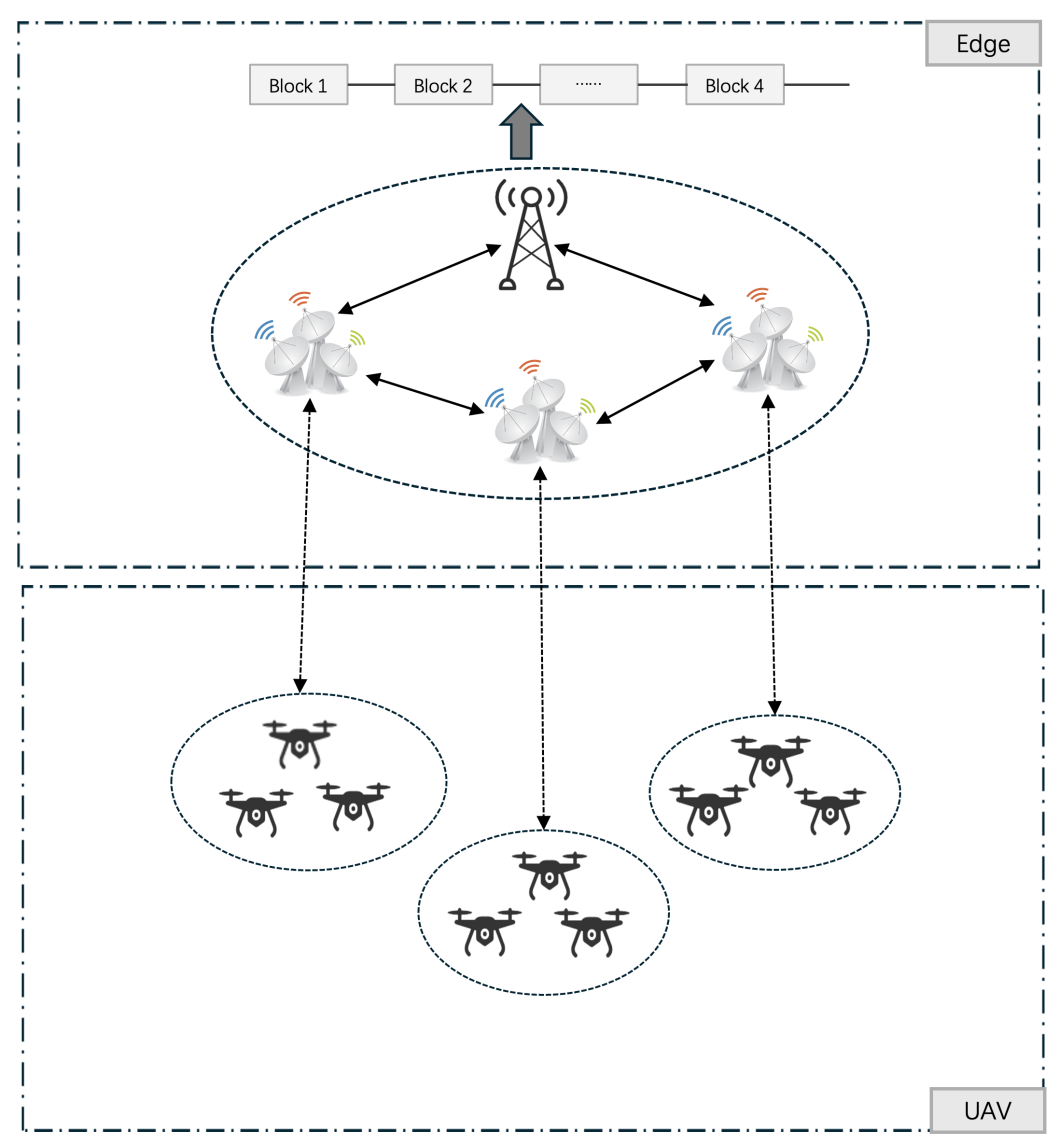
The interactions and data processing mechanisms between unmanned aerial vehicles and edge computing systems.

The integration of blockchain technology into UAV networks offers promising solutions to address inherent challenges. Blockchain’s decentralized architecture, immutability, and robust security mechanisms provide a foundation for enhancing the reliability and integrity of UAV communications, as shown in Figure 1. Within this context, adaptive sharding emerges as a critical technique to improve block efficiency, enabling rapid data processing and bolstering security measures, thus presenting a paradigm shift in the capabilities of aerial networks.

However, the implementation of traditional blockchain systems in UAV networks faces significant obstacles. These challenges primarily stem from the high mobility of UAVs and their complex, dynamic operational environment, resulting in sluggish node communication and prolonged data processing times. Current sharding technologies demonstrate limited adaptability to the unique dynamics of UAV networks. Consequently, there is an urgent need for a novel sharding paradigm capable of swiftly adjusting to node variations within the volatile drone ecosystem, thereby enhancing the overall performance and resilience of the blockchain system.

This research addresses three key questions: How can blockchain sharding be optimized for highly mobile UAV networks while maintaining security? What strategies best balance processing efficiency with network security? Furthermore, how can deep reinforcement learning enhance sharding performance in UAV networks?

In this paper, we hypothesize that an adaptive sharding mechanism, driven by deep reinforcement learning, will significantly improve both the processing efficiency and security of blockchain-enabled UAV networks. Specifically, we expect our dynamic sharding strategy to achieve higher transaction throughput compared to traditional approaches while maintaining system security and reducing consensus delay by at least 25%.

Overall, this study proposes an innovative adaptive sharding strategy specifically designed for UAV networks, leveraging blockchain technology. Through a comprehensive analysis of the UAV network environment and data transmission characteristics, we develop a refined model that addresses the unique challenges of aerial systems. Furthermore, we present an enhanced version of the Asynchronous Advantage Actor–Critic (A3C) algorithm, tailored to accommodate the dynamic nature and inherent uncertainties of UAV networks.

To validate the efficacy of the proposed strategy in improving data processing efficiency and ensuring system security, we conduct extensive simulation experiments. The results of these simulations underscore the potential of our innovative approach in optimizing UAV network operations. This research contributes to the growing body of knowledge at the intersection of UAV technology and blockchain systems, offering insights into the development of more efficient and secure aerial network infrastructures. The primary contributions of this study are as follows:

- We propose an adaptive sharding model optimized for UAV networks, offering a new solution for enhancing data security and processing efficiency within these networks.
- We perform a detailed analysis of data transmission rates to improve the model’s accuracy and applicability.
- We enhance the A3C algorithm to address the fragmentation challenges present in UAV networks.

The remainder of this paper is structured as follows: Section 2 offers an overview of related work; Section 3 outlines the system scenario and framework; Section 4 describes the performance analysis metrics; Section 5 defines the problem addressed in this study; Section 6 provides experimental results and analysis; and finally, conclusions are drawn in Section 7.

## 2. Related Works

In this section, we review the existing literature pertaining to intelligent vehicle networking technology, adaptive sharding, and the application and optimization of the A3C algorithm.

### 2.1. Intelligent Vehicle Networking Technology

The rapid advancement of Internet of Things (IoT) and Internet of Vehicles (IoV) technologies is progressively actualizing intelligent transportation systems. By integrating vehicles with technologies such as edge computing and blockchain, IoV technology presents novel opportunities for traffic management, vehicle safety, and the optimization of the driving experience. The following review examines pertinent research in the domain of IoV technology, highlighting several critical areas of inquiry.

Initially, regarding the safety task offloading strategy in vehicular networks, one study introduced a method based on dual deep Q-networks [1]. This innovative approach not only optimizes decision-making processes for task offloading but also integrates blockchain technology to ensure the security and integrity of data.

In the realm of privacy protection, another study developed a privacy-preserving computation offloading method within edge computing for connected vehicles [2]. This method utilized inter-vehicle communication (V2V) to establish routing, employing the Non-dominated Sorting Genetic Algorithm II (NSGA-II) to achieve multi-objective optimization while addressing privacy concerns.

Further research [3] has concentrated on the allocation of computing resources within the IoV in an edge computing environment, utilizing deep reinforcement learning to tackle resource allocation challenges in high real-time MEC systems, thereby significantly enhancing computing efficiency and response times.

To bolster the reliability of IoV systems, a study proposed a reliable computation offloading method for software-defined IoV [4]. This method optimized the computation offloading process through Software Defined Network (SDN) technology, thereby improving the reliability and flexibility of the system.

Addressing the issue of average energy efficiency in IoV networks, another study [5] conducted a comprehensive analysis and proposed a learning-based maximization method. By employing a learning approach, energy efficiency in IoV networks can be optimized.

Lastly, a study [6] investigated the challenges and future directions of artificial intelligence (AI), blockchain, and vehicular edge computing within the context of smart and secure IoV. The findings indicated that the convergence of AI, blockchain, and MEC technologies could yield smarter and safer services for IoV; however, it also highlighted challenges related to technology integration, privacy protection, and security.

In conclusion, research in IoV technology is advancing towards enhancing computational efficiency, optimizing resource allocation, and improving system reliability and security. Concurrently, as new technologies continue to emerge, the field of IoV must address critical issues such as multi-technology integration, data security, and privacy protection.

### 2.2. Adaptive Sharding

Blockchain technology [7] has garnered significant interest across various sectors due to its decentralized nature, transparency, and resistance to tampering. However, scalability issues have remained a critical barrier to its broader adoption. To address these limitations, adaptive sharding technology has emerged as a promising solution aimed at enhancing the scalability and performance of blockchain systems by dynamically adjusting network segments.

A study [8] presented a method of sharding to boost transaction throughput in blockchain systems. This research enhanced the performance of the Byzantine consensus protocol, thereby improving the throughput within individual shards. Additionally, SusChain et al. considered the unique aspects of drone networks, such as the high mobility of drones and the dynamic nature of the network. By optimizing sharding strategies and consensus mechanisms, their scheme achieves low energy consumption and high-throughput data dissemination.

Another study proposed SkyChain [9], a blockchain framework that employs deep reinforcement learning for dynamic sharding. Through an adaptive ledger protocol and intelligent sharding strategies, SkyChain optimizes the balance between system performance and security in dynamic environments.

RapidChain [10] is the first public blockchain protocol capable of tolerating Byzantine faults in up to one-third of its nodes. Through innovative technologies such as block pipelining and a secure reorganization mechanism, it achieves efficient data processing and complete fragmentation without a trusted setup.

Other research efforts have utilized Hidden Markov Models to implement dynamic sharding in blockchains, adapting to the collaborative demands within IoT ecosystems [11]. Conversely, dynamic sharding-based blockchain federated learning employs deep reinforcement learning to optimize sharding strategies, thereby enhancing blockchain performance and security in distributed learning tasks [12].

Overall, adaptive sharding technology markedly improves system processing capacity and response times by dynamically adjusting the blockchain network structure. This advancement opens up possibilities for broader applications of blockchain technology. Research in this area demonstrates the potential of sharding in boosting blockchain scalability and provides valuable guidance for future advancements in blockchain development.

### 2.3. Application and Optimization of A3C Algorithm

The integration of edge computing with blockchain technology offers innovative perspectives and solutions for resource management challenges. As a deep reinforcement learning technique, the A3C algorithm demonstrates substantial potential in this domain. By intelligently adjusting resource allocation strategies, the A3C algorithm facilitates optimal resource management across diverse network environments. For instance, Miaojiang et al. [13] proposed a learning-based framework for virtualized mobile vehicle services, addressing resource management issues in connected vehicle networks via the A3C algorithm, which enhances network performance through dynamic resource allocation.

The A3C algorithm also plays a pivotal role in the convergence of blockchain and mobile edge computing. Jianbo et al. [14] explored the use of blockchain for mining tasks in mobile systems, proposing an approach for task offloading and resource pricing and allocation through MEC and the A3C algorithm to maximize miner profitability. Similarly, Yexinyu et al. [15] integrated blockchain into the Internet of Vehicles (IoV) to ensure the accuracy and reliability of data transmissions. They developed an intelligent framework for computing and caching resource allocation, employing the A3C algorithm to optimize these processes and enhance overall system performance.

The versatility of the A3C algorithm in optimizing resource management in edge computing environments with blockchain technology is highlighted in recent studies. It offers valuable insights for intelligent resource pricing and allocation in various settings, such as vehicular networks, cloud-supported edge user scenarios, and blockchain systems enhanced by MEC. The algorithm shows promise in revolutionizing resource management strategies in these contexts.

In this study, we introduce an adaptive sharding strategy specifically designed to align with the unique characteristics of drone networks, with the objective of enhancing data processing efficiency and system security. Furthermore, we formulate the challenges of video offloading and resource allocation as a Markov Decision Process (MDP) and investigate potential solutions through deep reinforcement learning techniques, all while preserving a decentralized control framework. This research seeks to advance drone network technology and offers valuable insights for integrating blockchain technology, aiming to establish a robust connection between blockchain systems and drone networks.

Based on the work of Xie et al. [16] and Yuan et al. [17], this paper introduces innovative solutions for intelligent resource allocation, adaptability in dynamic environments, and security in UAV networks. We developed a refined adaptive sharding model and an enhanced A3C algorithm to address the specific challenges and uncertainties of UAV networks. These advancements offer new perspectives and significant improvements for the future of UAV technology and intelligent transportation systems.

## 3. System Scenario and System Framework

### 3.1. System Scenario

In this section, the video offloading decision in the system model UAV network scenario is proposed, including the UAV network system to generate transactions, the MEC system video analysis platform to generate transactions, and the blockchain system transactions. The model of the three parts is designed as follows.

In this study, we examine a blockchain-enabled unmanned aerial vehicle network within the context of the IoT. The network is structured around a macro-cell base station (MBS) situated at the center of the designated area, with multiple roadside units (RSUs) strategically distributed around the MBS and interconnected via wired connections. This paper presents a framework for facilitating interference-free data transmission through the utilization of orthogonal spectrum. Let M represent the set of RSUs. Consequently, we define the set of UAVs as N, with each UAV*n* being associated with its corresponding RSU*m* for the purpose of service provision. Given the relatively limited computational capabilities of the UAVs, the integration of a mobile edge computing server is essential for executing video analysis tasks, thereby enhancing the quality of the user’s computing experience. In essence, this system enables UAVs to utilize the MEC server for video analysis, ultimately improving the overall user experience in terms of computational efficiency.

In the blockchain framework, all resource sharing units constitute the nodes of the blockchain. These nodes fulfill two distinct functions: they can operate as either standard nodes or consensus nodes. The primary objective of the blockchain system is to manage transactions originating from the mobile edge computing network, which consist of records pertaining to video offloading data. To achieve this objective, the blockchain system must execute two critical steps: block generation and the consensus process. Standard nodes are tasked with the transmission and reception of ledger data, whereas consensus nodes are responsible for the generation of blocks and the execution of the consensus process. Let K represent the set of consensus nodes, from which we select *K* consensus nodes from a total of *M* nodes based on specific criteria. The UAV network’s Achilles’ heel lies in its uplink capacity, a constraint that stifles the scalability of video offloading endeavors due to MEC servers’ inherent resource limitations. Here, the cellular system’s uplink communication channel emerges as a vital conduit, enabling Vehicle-to-Infrastructure (V2I) communication, where UAVs transmit video data seamlessly to roadside units. Given this backdrop, optimizing both network and computational resources becomes a strategic imperative. This study, therefore, incorporates a nuanced consideration of uplink MEC, recognizing its pivotal role in maximizing resource utilization and enhancing the overall performance of the UAV-based video offloading system. By doing so, we strive to unlock the full potential of this innovative technology, paving the way for more efficient, scalable, and secure video transmission in UAV networks.

To enhance system performance, the blockchain architecture employs a hierarchical and sharding approach, segmenting the system into a preliminary consensus group (PG) and a final consensus group (FG). The final consensus group is supported by a Trusted Execution Environment (TEE). The preliminary consensus group PG consisting of $K_{1}$ members, is responsible for achieving initial transaction consensus, subsequently packaging the validated transactions into a micro-block, which is then transmitted to the FG. The FG receives the micro-block from the PG, consolidates it into a larger block, and finalizes the consensus process.

Moreover, edge caching ascends as a vital component, ensuring seamless on-demand video streaming amidst cellular networks. Within our study’s scope, the video analysis process involves crucial stages—decoding, preprocessing, and object detection—harmoniously orchestrated within the UAV network realm. We strive towards harmonizing network and computational resources, catapulting blockchain system throughput while, in parallel, truncating mobile edge computing system latencies. By strategically positioning RSUs with innate MEC might, we carve out a robust platform to embark on the exhilarating journey of video analysis optimization.

### 3.2. System Framework

#### 3.2.1. MEC System

**Figure 2:**
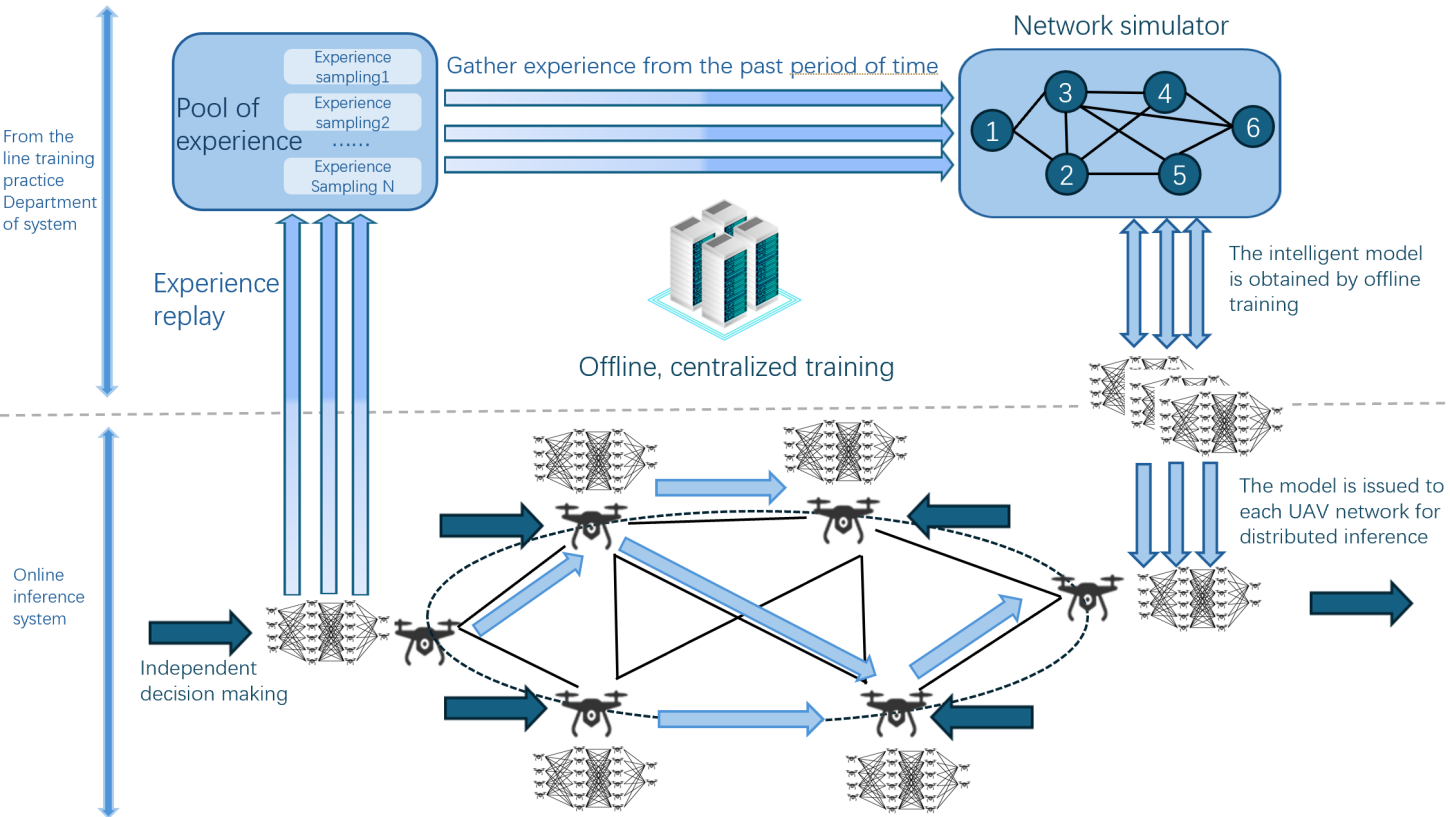
UAV network model training and deployment flowchart.

**Figure 3:**
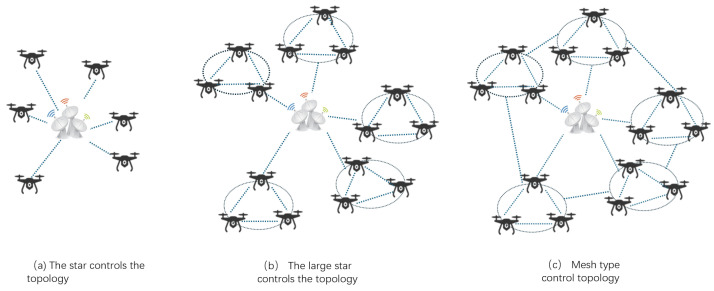
UAV network topology control structure diagram.

As shown in Figure 2 and Figure 3, in the context of offloading tasks for video analysis applications within mobile edge computing systems, we delineate two sets, denoted as $P_{nm}\left(t\right)$ and $h_{nm}\left(t\right)$. Specifically, $P_{nm}\left(t\right)$ signifies the transmit power from UAV*n* to RSU*m* at a given time *t*, while $h_{nm}\left(t\right)$ denotes the associated channel gain. The system bandwidth is established at *B* Hz. $N_{0}$ represents the noise power, and $I_{m}\left(t\right)$ indicates the received aggregated interference. Our focus then shifts towards elucidating the transmission interference at *t*. Based on this theoretical backdrop, we articulate the transmission rate from UAV*n* to RSU*m*, capturing the intricate interplay between power, channel gain, bandwidth, noise, and interference.

(1) $$R_{nm}\left(t\right)=B\log_{2}\left(1+\frac{P_{nm}\left(t\right)h_{nm}\left(t\right)}{N_{0}+I_{m}\left(t\right)}\right)$$

The transmission delay associated with the offloading of each video datum is defined with $D_{nm}^{\mathrm{tr}}\left(t\right)$ and can be articulated as

(2) $$D_{nm}^{\mathrm{tr}}\left(t\right)=\frac{L}{R_{nm}\left(t\right)}$$

where *L* (in bits) is the size of each offloaded video datum.

According to the literature [16], the energy consumption associated with the offloading of video data from a UAV*n* to an RSU*m* can be articulated as follows:

(3) $$E_{nm}^{\mathrm{tr}}\left(t\right)=P_{nm}\left(t\right)D_{nm}^{\mathrm{tr}}\left(t\right)$$

The execution of video analysis tasks on the mobile edge computing server necessitates substantial computational resources. We define $f_{m}$ (in cycles per second) as the CPU cycle frequency required by the *m*-th MEC server, while *c* (in cycles per bit) represents the number of CPU cycles needed to process a single bit of video data. Consequently, as indicated in the literature [16], the computation delay for MEC server *m* when executing the task for vehicle *n* can be articulated as follows:

(4) $$D_{nm}^{\mathrm{comp}}\left(t\right)=\frac{Lc}{f_{m}}$$

Let κ denote the computational energy efficiency factor of the processor. According to the findings presented in the literature [18], the energy consumption associated with video analysis on MEC server *m* can be formulated as follows:

(5) $$E_{nm}^{\mathrm{comp}}\left(t\right)=\kappa cf_{m}^{2}L$$

The *m*-th MEC server’s total delay for video analysis includes both transmission and computation delays. The formula from the literature [16] defines this overall delay:

(6) $$D_{nm}^{\mathrm{total}}\left(t\right)=D_{nm}^{\mathrm{tr}}\left(t\right)+D_{nm}^{\mathrm{comp}}\left(t\right)$$

In a similar vein, the total energy consumption $E_{nm}^{\mathrm{total}}\left(t\right)$ can be expressed as follows, in accordance with the literature [16]:

(7) $$E_{nm}^{\mathrm{total}}\left(t\right)=E_{nm}^{\mathrm{tr}}\left(t\right)+E_{nm}^{\mathrm{comp}}\left(t\right)$$

#### 3.2.2. Blockchain System

Scalability is a critical factor that defines the capacity of a network, encompassing various elements such as the number of nodes, the volume of transactions that can be processed, and the processing speed of the network. Transactions are submitted to the blockchain system, where block producers are selected based on a predetermined scheduling policy. These block producers are responsible for generating blocks by collecting, verifying, and packaging transactions.

As indicated in the literature [16], transaction throughput TP is influenced by block size BS, block interval BI, and average transaction size TS, with the relationship articulated as follows:

(8) $$TP=\frac{BS}{BI\times TS}$$

Consequently, transaction throughput can be enhanced by either increasing the block size or reducing the interval. It is important to note, however, that the scalability of a blockchain system is also influenced by additional factors such as latency, security, and decentralization.

Delegated Proof-of-Stake (DPoS) blockchains must navigate a trade-off between security and decentralization while exhibiting faster performance compared to Proof-of-Work (PoW) and Proof-of-Stake (PoS) blockchains. The operational procedure of a DPoS blockchain involves two primary steps: (1) the selection of *K* block producers from a pool of candidates and (2) the signing of the first block by the *i*-th block producer until *i* equals *K*. Thus, the consensus latency in DPoS encompasses the time required for block propagation and verification. Let $r_{ij}$ represent the wire transmission rate from the *i*-th block verifier to the *j*-th block verifier, where i,j∈{1,2,…,K} are defined. Consequently, the propagation time can be derived from the literature [16] as follows:

(9) $$T_{p}=\sum_{i=1}^{K}\frac{BS}{r_{ij}}$$

According to the literature [16], in terms of block verification, the computational cost associated with the encryption operation is taken into account. Let $f_{i}$ denote the CPU cycle frequency necessary for the *i*-th verifier to validate a block, and let *c* represent the number of CPU cycles required for this verification. The verification time can then be expressed as follows:

(10) $$T_{v}=\sum_{i=1}^{K}\frac{c}{f_{i}}$$

The effectiveness of a blockchain system can be measured by its Delay to Finality (DTF) metric, which reflects the time it takes for transactions to become irreversible on the blockchain. This includes the duration for generating, propagating, and verifying blocks. DTF is a crucial factor in determining the overall performance and reliability of a blockchain network:

(11) $$DTF=BI+T_{p}+T_{v}$$

In Intelligent and Autonomous Vehicle (IoAV) networks, it is common for vehicles to seek the final results of transactions within a minimal time frame. It is defined that a block must be issued and verified across several consecutive block intervals, denoted as λ (where λ>1). Therefore, according to the literature [16], the DTF must adhere to the following constraints:

(12) DTF≤λBI

Blockchain security is crucial for protecting against outside threats and preventing manipulation. Blockchain systems face multiple potential attacks, including double spending, Sybil attacks, DDoS attacks, and 51% attacks. Therefore, in order to ensure system security, we need to limit the number of malicious verifiers *F*, where *F* constraints can be obtained according to the literature [16]:

(13) $$F<\left\lfloor \frac{K-1}{3}\right\rfloor$$

For unmanned aerial vehicle networks, decentralization refers to the diversity of ownership, influence, and value within the blockchain. In a blockchain network, all transactions and blocks are broadcast, verified, and recorded among all participants in a decentralized manner. As long as more than half of the computational resources remain honest, the system is immutable, stable, and resistant.

Scalability becomes a major concern when applying blockchain to UAV networks. First, the transaction throughput can be improved by adjusting the block size or reducing the block interval. Second, reducing the block interval imposes a stricter constraint on the consensus latency.

Therefore, in the context of UAV networks, it is necessary to carefully adjust the block size, adjust the block interval, or select the block generator and consensus algorithm to achieve a balance of blockchain scalability.

#### 3.2.3. Consensus Models

Practical Byzantine Fault Tolerance (PBFT) is adopted as the consensus algorithm in this paper due to its good security in asynchronous networks. The consensus process consists of five phases: request, prepare, prepare, submit, and reply. Specifically, a client node issues a large block, and other nodes audit and compare with each other, then finally reply with the audit result and signature to the client node.

## 4. Performance Analysis Indicators

### 4.1. Sharding Security

Blockchain security is fundamental in ensuring resilience against tampering by malicious nodes, particularly through the consensus mechanism. This characteristic is crucial for assessing the overall performance of a blockchain system. The consensus algorithm discussed in this paper is PBFT, which operates under an adversarial model defined by the condition $f<\frac{n}{3}$, where *n* represents the total number of consensus nodes and *f* signifies the number of malicious nodes. This stipulation indicates that the fraction of malicious nodes within the network must not exceed one-third. Furthermore, in partitioned blockchain systems, security is also contingent upon the number of partitions present. By striking a balance between the number of malicious nodes and total consensus nodes, as well as understanding how partitions impact security, stakeholders can enhance the security measures of blockchain systems to mitigate potential vulnerabilities.

#### 4.1.1. Partition Security

We categorize the partitioned group *G* into *m* subsets, each comprising *n* nodes. It is assumed that each node possesses a probability of being malicious, denoted as *p*. We define the discrete random variable *X* to represent the count of malicious nodes within the partition *i*. The security probability of $G_{i}$ is quantified as $P_{s}\left(G_{i}\right)$, where i∈{1,2,…,m}. According to the findings presented in the literature [17], $P_{s}\left(G_{i}\right)$ can be articulated as follows:

(14) Ps(Gi)=∑k=0⌊n3⌋−1nkpk(1−p)n−k

Observational analysis reveals that partition security is a monotonically decreasing function of *m*.

#### 4.1.2. Global Security

The significance of a node within the blockchain system plays a crucial role in determining the overall security probability. By evaluating the security probabilities of individual shards, we can gauge the global security of the blockchain system. Based on the literature [17], we can derive the following conclusions:

(15) $$P_{s}\left(G\right)=\prod_{i=1}^{m}P_{s}\left(G_{i}\right)$$

To ensure the security of the proposed blockchain system, we impose security constraints on the blockchain system $P_{s}\left(G\right)\geq P_{s}^{\min}$, where $P_{s}^{\min}$ represents the security parameter.

### 4.2. Transaction Confirmation Time

Transactions undergo an initial verification process conducted by the PG, $K_{1}$, to achieve preliminary consensus. Subsequently, these transactions are organized into micro-blocks and transmitted to the FG, where they are ultimately consolidated into larger blocks to finalize the consensus.

#### 4.2.1. Processing Time of $PG_{i}$

The consensus time encompasses the packaging time, consensus time, and delivery time.

The packaging duration for $PG_{i}$ can be derived from the literature [7] as follows:

(16) $$T_{1}^{p}=\frac{S_{m}}{C_{1}R_{1}}$$

In this equation, $S_{m}$ denotes the size of the micro-block, $C_{1}$ represents the computational resources necessary for processing the micro-block per unit size, and $R_{1}$ indicates the computational resources allocated to the $PG_{i}$.

The consensus duration for $PG_{i}$ can be computed as follows, where $r_{1}$ signifies the transmission rate of $PG_{i}$. According to literature [19]:

(17) $$T_{1}^{c}=\frac{3K_{1}S_{m}}{r_{1}}$$

As per literature [19], the transaction time for $PG_{i}$ is expressed as

(18) $$T_{1}^{t}=\frac{S_{m}}{r_{2}}$$

In this context, $r_{2}$ represents the transmission rate between $PG_{i}$ and FG, leading to the total transaction processing time for PG being articulated as

(19) $$T_{1}=T_{1}^{p}+T_{1}^{c}+T_{1}^{t}$$

#### 4.2.2. Processing Time of FG

The overall transaction processing time for the FG comprises the block generation time and the final consensus time.

The FG is responsible for assembling the micro-blocks received from the PG into larger blocks. The time required for this process is denoted as $T_{G}$. According to the literature [19], the final consensus time can be approximated as follows:

(20) $$T_{2}^{c}=\frac{3K_{2}S_{b}}{r_{3}}$$

Here, $r_{3}$ represents the transmission rate among consensus nodes within FG, allowing the total transaction processing time for FG to be described as follows, as per the literature [19]:

(21) $$T_{2}=T_{G}+T_{2}^{c}$$

#### 4.2.3. Throughput Analysis

Throughput is defined as the number of transactions processed per second by a blockchain system. According to the literature [19], it can be calculated using the following formula:

(22) $$TP=\frac{mS_{m}}{T_{1}+T_{2}}$$

In this equation, *m* represents the number of partitions, $T_{1}+T_{2}$ denotes the bulk generation time, and $S_{m}$ indicates the size of the micro-block.

## 5. Problem Formulation

In this section, the concepts of partition security, block generation interval, and micro-block size are integrated into a joint optimization framework aimed at enhancing both the throughput and the security of the blockchain system. To address this non-convex optimization challenge, we utilize a third-order deep reinforcement learning (DRL) algorithm.

The issue of video offloading and resource allocation is approached as a discrete Markov Decision Process (MDP) aimed at maximizing system rewards. In this scenario, unmanned aerial vehicles are tasked with selecting which videos to offload and efficiently distributing limited resources to enhance video quality and user satisfaction. By considering the current state, UAVs can strategically allocate resources to optimize system rewards. This methodology ensures that resources are utilized effectively to improve overall system performance and user experience, ultimately leading to enhanced video quality and satisfaction levels.

DRL algorithms like Asynchronous Actor–Critic Agents excel in solving problems with both discrete and continuous action spaces, particularly in parallel training settings. These algorithms are adept at addressing sequential decision-making challenges in uncertain environments by optimizing reward functions, ensuring swift convergence, and enabling quick training on CPUs. With DRL, UAV entities can reduce communication overhead and improve network security and resilience. By leveraging local data to determine the best course of action, DRL algorithms allow for efficient and effective decision-making in dynamic environments. This makes them a valuable tool for optimizing resource allocation and enhancing overall system performance.

### 5.1. The Joint Optimization Problem

#### 5.1.1. State Space

The state space of the blockchain system is defined as $S=\{s_{t}|t=1,2,\ldots \}$, where $s_{t}$ denotes the state of the blockchain system at the *t*-th time. This state encompasses the transmission rate of the wireless link, denoted as $r_{t}$, and the computational capacity of the blockchain node, represented as $c_{t}$. Consequently, $s_{t}$ can be articulated as follows, in accordance with reference [19]:

(23) $$s_{t}=\left\{r_{t},c_{t}\right\}$$

#### 5.1.2. Action Space

To enhance both the throughput and partition security of the blockchain system, it is necessary to adjust the number of partitions, the block generation interval, and the micro-block size at each time period *t*. Therefore, the action space is defined as $A=\{a_{t}|t=1,2,\ldots \}$, where $a_{t}$ can be expressed as per the literature [18].

(24) $$a_{t}=\left\{m_{t},\tau_{t},S_{m,t}\right\}$$

#### 5.1.3. Reward Function

This study aims to optimize partition security and throughput; hence, the reward function is formulated as follows.

(25) $$R\left(s_{t},a_{t}\right)=\left\{\begin{array}{cc} r(s_{t},a_{t}), & \mathrm{if}\;C_{1}\cap C_{2}\cap C_{3}\\ -\infty , & \mathrm{otherwise} \end{array}\right.$$

In this context, $C_{1}$ ensures that the micro-block size remains within the specified upper limit, $C_{2}$ guarantees that the confirmation time does not exceed the generation time for a predetermined number of blocks, and $C_{3}$ safeguards the security of each blockchain partition. Furthermore, the reward function $V^{\pi }\left(s_{t}\right)$ signifies the long-term reward and can be computed as follows:

(26) $$V^{\pi }\left(s_{t}\right)=E_{\pi }\left[\sum_{k=0}^{\infty }\gamma^{k}r\left(s_{t+k},a_{t+k}\right)|s_{t}\right]$$

Here, γ denotes the discount rate, while $r(s_{t},a_{t})$ represents the immediate benefit, defined as the weighted sum of the global security and throughput of the blockchain system.

(27) $$r\left(s_{t},a_{t}\right)=\alpha P_{s}\left(G\right)+\left(1-\alpha \right)TP$$

Subsequently, we employ the adoption-based Deep Q-Network (DQN), Asynchronous Actor–Critic, and Deep Deterministic Policy Gradient (DDPG) methodologies to address the optimization problem. In the context of DDPG, both the action space and the state space are continuous.

#### 5.1.4. Complexity Analysis

To evaluate the complexity of the proposed scheme, we first analyze the dimensions of the state space. The variables $r_{t}$ and $c_{t}$ within the state space $s_{t}$ possess $d_{r}$ and $d_{c}$ distinct discrete states, respectively. Therefore, the size of the state space is $O(d_{r}d_{c}N)$, where *N* represents the total number of nodes.

Next, we examine the implications of different algorithms. In the case of the two-way DQN, it utilizes a single network, allowing actions to be represented as functions of states. Consequently, the complexity is O(|S|·|A|).

### 5.2. Video Offloading and Resource Allocation Challenges

Within the framework of unmanned aerial vehicle systems, the problem of resource allocation (RA) based on DRL can be articulated as an MDP. This formulation encompasses the definition of the state space S, action space A, state transition probabilities P, and the reward function R pertinent to the system. The application of DRL methodologies enables UAV entities to mitigate communication overhead while enhancing the security and resilience of the network, all while locally observing and deriving the optimal policy.

#### Markov Decision Process

The state space at decision time *t* is characterized as the amalgamation of wireless channel conditions $H_{t}$, the computational capacity $F_{t}$ of the mobile edge computing system, and the average transaction size $TS_{t}$, which can be represented as follows:

(28) $$s_{t}=\left\{H_{t},F_{t},TS_{t}\right\}$$

The action space encompasses the offloading decision $x_{t}$, power allocation $P_{t}$, block size $BS_{t}$, and block interval $BI_{t}$. Consequently, the action space at decision time *t* is denoted as follows:

(29) $$a_{t}=\left\{x_{t},P_{t},BS_{t},BI_{t}\right\}$$

where the block producer is represented by *k*, k∈K. Here, $x_{t}=1$ indicates the selection of MEC server *m* for task offloading, while $x_{t}=0$ signifies the opposite. Furthermore, $BS_{max}$ and $BI_{max}$ represent the block size limit and the maximum block interval, respectively, where $BS_{t}\leq BS_{max}$ and $BI_{t}\leq BI_{max}$ are the corresponding designations.

In UAV systems, the resource allocation challenge associated with video analysis tasks is influenced by factors such as data size, CPU cycles, and latency. The reward function typically incorporates considerations of video resolution and Quality of Experience (QoE) to facilitate resource allocation. In this context, the focus is on optimizing the transaction throughput of the blockchain system while minimizing latency within the MEC framework. The total delay experienced by the MEC system comprises both transmission and computation delays, with transmission delay being contingent upon data size and computation delay being a function of both data size and CPU cycles. In a blockchain context, throughput is determined by block size, block interval, and average transaction size. Given that UAVs generally aim to receive transaction outcomes promptly, the joint optimization problem is articulated as a reward function within the UAV system.

(30) $$R\left(s_{t},a_{t}\right)=\left\{\begin{array}{cc} r(s_{t},a_{t}), & \mathrm{if}\;C_{1}\cap C_{2}\cap C_{3}\\ -\infty , & \mathrm{otherwise} \end{array}\right.$$

(31) $$C_{1}:E_{nm}^{total}\left(t\right)\leq E_{max}$$

(32) $$C_{2}:\sum_{n=1}^{N}x_{nm}\left(t\right)f_{nm}\left(t\right)\leq F_{m}^{max}$$

The parameters α and β serve to weight the objective function and mapping factor, respectively, ensuring that the objective function is uniformly scaled. $E_{max}$ and $F_{m}^{max}$ denote the cumulative maximum energy consumption and total computational capacity of the MEC server, respectively. Thus, the reward function can be expressed as follows:

(33) $$r\left(s_{t},a_{t}\right)=\alpha TP+\left(1-\alpha \right)\beta \left(E_{max}-E_{nm}^{total}\left(t\right)\right)$$

### 5.3. Deep Reinforcement Learning Approach

The Asynchronous Actor–Critic algorithm maintains both a policy function $\pi_{\theta }\left(a_{t}|s_{t}\right)$ and a value function $V_{w}\left(s_{t}\right)$, with θ and *w* representing weight parameters. The policy and value functions undergo updates following each action taken over a time horizon *T*. This mechanism informs the agent regarding which actions yield rewards and which incur penalties. According to the literature, the value of the discounted reward $R_{t}$ can be defined as follows:

(34) $$R_{t}=\sum_{k=0}^{T-1}\gamma^{k}r_{t+k}$$

In the Asynchronous Actor–Critic Agents framework, a discount factor, denoted as γ, is employed to modulate the significance of future rewards. A higher discount factor results in a diminished weighting of future rewards, as these rewards are subject to greater discounting. In this study, we established the discount factor at 0.99. As indicated in the literature [18], the value of a state $s_{t}$ under a specific policy π can be articulated as the expected value of the future rewards that the agent can accrue through the application of policy π in that state, with the objective of maximizing immediate rewards.

(35) $$V^{\pi }\left(s_{t}\right)=E_{\pi }\left[\sum_{k=0}^{\infty }\gamma^{k}r_{t+k}|s_{t}\right]$$

Furthermore, the advantage function can be derived as outlined in the literature [19].

(36) $$A\left(s_{t},a_{t}\right)=Q\left(s_{t},a_{t}\right)-V\left(s_{t}\right)$$

Given that the value function $V(s_{t})$ for state $s_{t}$ cannot be directly ascertained within the A3C algorithm, the discounted return *r* serves as an approximation of $V(s_{t})$, which is utilized to compute the advantage estimate. The literature [19] provides a formulation for estimating the advantage.

(37) $$A\left(s_{t},a_{t}\right)\approx r_{t}+\gamma V\left(s_{t+1}\right)-V\left(s_{t}\right)$$

In terms of entropy, the loss function associated with the strategy (or player) is articulated as per the literature [18]:

(38) $$L_{\pi }=\log\pi_{\theta }\left(a_{t}|s_{t}\right)\left(R_{t}-V_{w}\left(s_{t}\right)\right)+\beta H\left(\pi_{\theta }\right)$$

The entropy measure, denoted as $H(\pi_{\theta })$, functions as a mechanism to encourage agents to eschew strategies characterized by lower uncertainty. The parameter β, referred to as the entropy coefficient, is instrumental in determining the relative significance of entropy in relation to reward; this coefficient is a hyperparameter that can be optimized through random search methodologies. Consequently, the literature [18] delineates the goal update for participants.

(39) $$\theta \leftarrow \theta +\alpha \nabla_{\theta }\log\pi_{\theta }\left(a_{t}|s_{t}\right)\left(R_{t}-V_{w}\left(s_{t}\right)\right)+\beta \nabla_{\theta }H\left(\pi_{\theta }\right)$$

The value function (critic) operates as an independent function. According to the literature [18], the advantage function, based on L2-loss, can be expressed in a specific manner:

(40) $$L_{v}={\left(R_{t}-V_{w}\left(s_{t}\right)\right)}^{2}$$

Thus, reviewers will adjust towards the goal, as articulated in the literature [18].

(41) $$w\leftarrow w-\alpha \nabla_{w}{\left(R_{t}-V_{w}\left(s_{t}\right)\right)}^{2}$$

Finally, as referenced in the literature [18], the standard uncentered RMSProp update is implemented within the A3C algorithm.

(42) $$g\leftarrow \beta g+\left(1-\beta \right){\left(\nabla \theta \right)}^{2}$$

The momentum parameter β is incorporated into the RMSProp optimization algorithm to mitigate oscillations and facilitate a more rapid convergence towards local minima along the X-axis. However, excessive momentum may result in uncontrolled fluctuations between local minima. Typically, the momentum is denoted by ρ and is generally set to 0.9, while the learning rate is denoted by α as a small positive ϵ number.

### 5.4. Explanation of Algorithm Innovation

As shown in Algorithm 1, the proposed A3C algorithm introduces several improvements and innovations over traditional A3C and other reinforcement learning algorithms. Firstly, it employs asynchronous parallel training, where multiple worker processes interact independently with the environment, updating the global network in real time. This approach avoids the waiting time issues between processes that are common in traditional synchronous methods. The asynchronous update mechanism accelerates model learning efficiency, significantly reducing training latency. This design not only increases the frequency and speed of training but also reduces blockages in the update of global model parameters.

Secondly, the proposed A3C algorithm eliminates the experience replay mechanism, opting for direct real-time policy updates instead. This avoids the memory burden associated with storing large amounts of historical data, ensuring the algorithm’s efficiency. The absence of a replay buffer means the algorithm continuously learns from policy data, leading to more robust and faster updates to the policy network. Complementing this, the asynchronous exploration of diverse state spaces by worker processes naturally reduces the variance in gradient updates, enhancing the stability of the model.

Moreover, the proposed algorithm optimizes both the policy network and the value network simultaneously, using a shared network architecture to estimate the probability distribution of actions and the state values. Sharing network parameters not only reduces computational overhead but also enhances the adaptability and convergence capabilities of the model when dealing with complex state spaces. Additionally, the algorithm extends beyond the limitations of traditional discrete action spaces, supporting optimization in continuous action spaces as well. See Algorithm 1.
**Algorithm 1** Asynchronous Advantage Actor–Critic.**Require:** S,A,MAX_EP,γ,UPDATE_GLOBAL_ITER,α,β,Env **Ensure:** Trained global policy and value networks
  1:Initialize global network θ, optimizer O, episode counter GEC, reward GR, queue *Q*  2:**function** Net(θ)  3:      Define network architecture, forward pass, action selection, and loss function  4:**end function**  5:**function** Worker(id)  6:      Initialize local network $\theta^{\prime }$ and environment env  7:      **while** GEC<MAX_EP **do**  8:            Reset env, initialize episode reward ep_r  9:            **while** episode not done **do**10:                Select action *a*, observe *r* and $s^{\prime }$, update ep_r11:                **if** local steps % UPDATE_GLOBAL_ITER==0 **then**12:                      Update global θ, synchronize $\theta^{\prime }$13:                **end if**14:           **end while**15:           Update GEC and GR, push (id,ep_r) to *Q*16:     **end while**17:**end function**18:Start multiple Worker processes19:Collect results, compute and report average reward

## 6. Experiment and Analysis

In this section, we evaluate the proposed partitioned blockchain scaling framework. The simulation experiments were conducted within a software environment using Python 3.6, specifically leveraging TensorFlow version 1.15.0 and PyTorch version 1.8.1. Additionally, the experimental setup included an NVIDIA GeForce RTX 2050 display adapter (GPU) and was supported by a CUDA version 12.2.147 driver.

**Figure 4:**
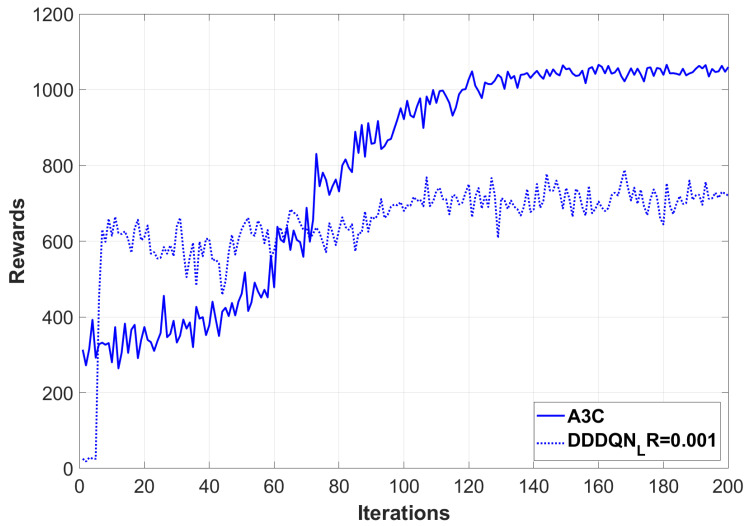
Comparison of Rewards obtained by A3C and DDDQN with learning rate 0.001 during training process.

Figure 4 illustrates the experimental results, which strongly demonstrate the superiority of the A3C algorithm within a UAV network environment. The algorithm exhibits both rapid convergence and the achievement of higher long-term rewards. These findings further corroborate the effectiveness of our proposed adaptive partitioning strategy in enhancing the performance of the blockchain system.

**Figure 5:**
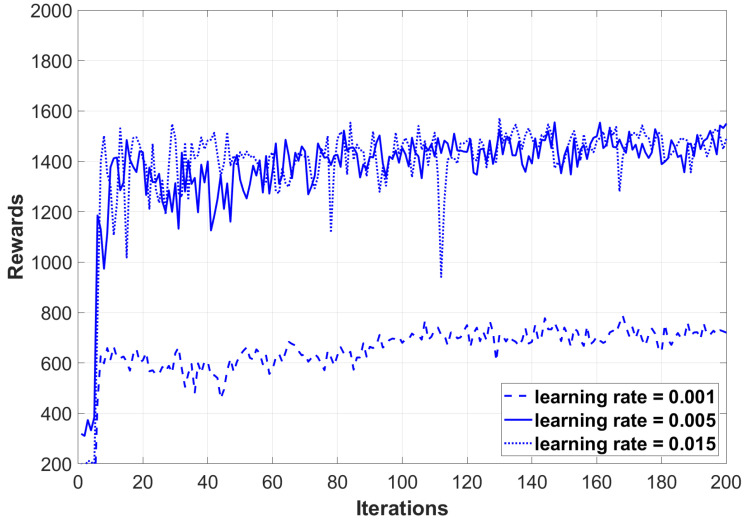
Comparing reward curves for different learning rates in DDDQN algorithm.

Figure 5 presents a comparison of the rewards obtained by the DDDQN (Dueling Double DQN) algorithm at different learning rates (0.001, 0.005, and 0.015) within a UAV environment. The results indicate that learning rates of 0.005 and 0.015 yield higher rewards, suggesting improved stability and more rapid convergence. Conversely, a learning rate of 0.001 leads to lower average rewards and greater volatility. These observations imply that within the specified range, increasing the learning rate can enhance the adaptability of the DDDQN algorithm in the UAV environment.

**Figure 6:**
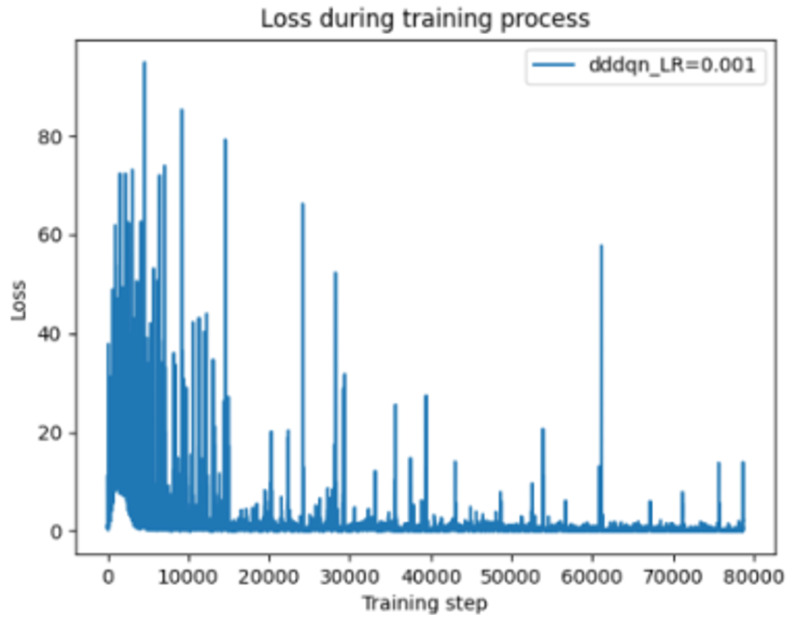
Change of loss function for DDDQN LR = 0.001.

Figure 6 demonstrates the training loss over time with a learning rate of 0.001. Initially, the loss decreases sharply, but the training process exhibits periodic fluctuations, indicating instability. Despite the overall trend of decreasing average loss, which suggests that the model is learning, the frequent oscillations point to stability issues at this learning rate.

**Figure 7:**
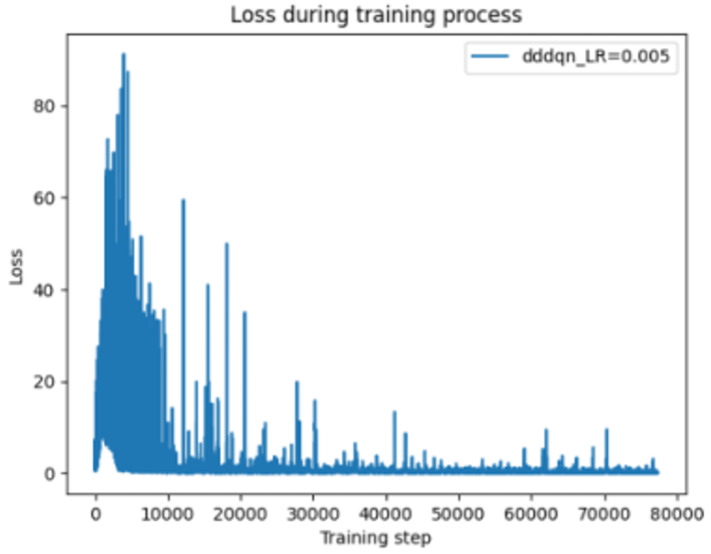
Change of loss function for DDDQN LR = 0.005.

Figure 7 illustrates that when the learning rate is increased to 0.005, the loss decreases more rapidly and with greater stability compared to a rate of 0.001. This finding is consistent with the conclusions drawn from Figure 1, indicating that a higher learning rate facilitates more efficient convergence of the model and helps avoid prolonged periods of high loss.

**Figure 8:**
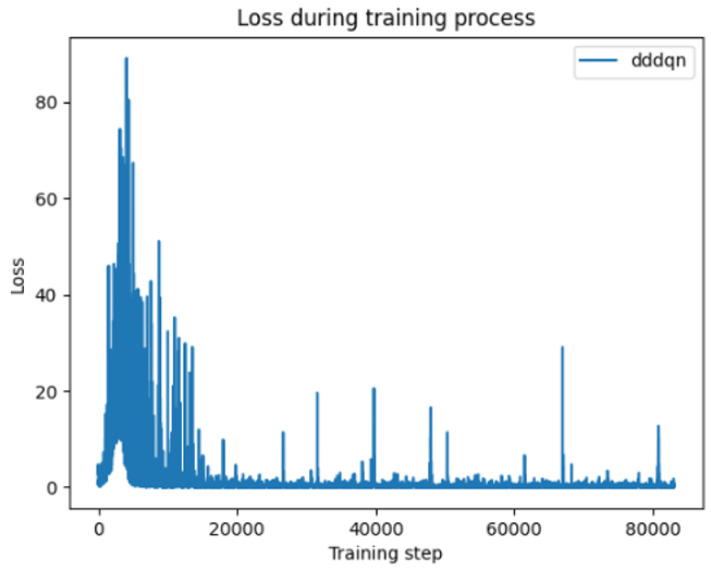
Change of loss function for DDDQN method.

Figure 8 depicts the variation in the loss function for the DQN algorithm with a learning rate of 0.1. Although the initial loss decreases, there is considerable overall fluctuation, with pronounced peaks particularly evident in the early stages of training. This may be attributed to the high learning rate causing excessive parameter updates, thereby impacting the model’s convergence.

**Figure 9:**
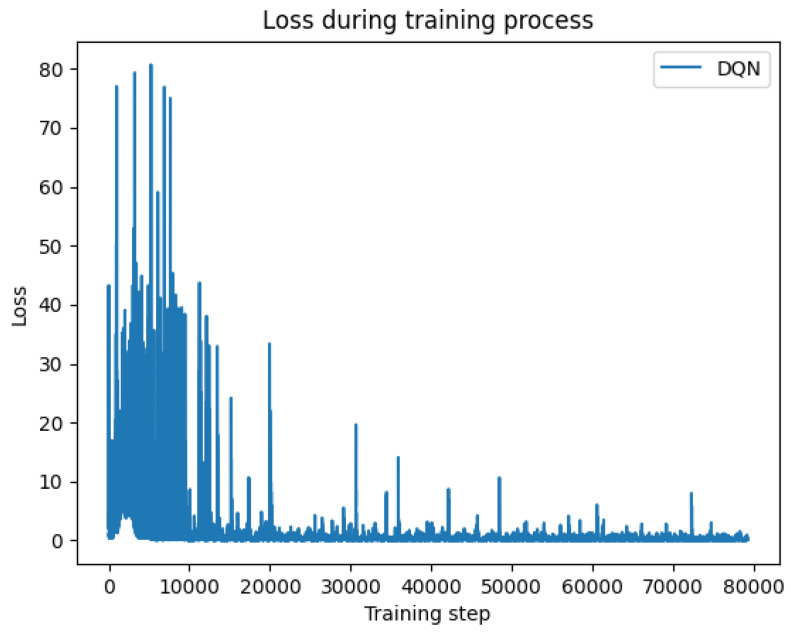
Change of loss function for DQN method.

Figure 9 illustrates the changes in the loss function for the DDDQN algorithm with a learning rate of 0.1. During the initial phases of training, the high learning rate leads to significant fluctuations in the loss function; however, the loss gradually decreases and stabilizes as training progresses. Nonetheless, compared to scenarios with lower learning rates, the loss function exhibits more pronounced variations, resulting in a less smooth training process.

The experimental results offer valuable insights for practical UAV network deployments. The superior performance of the A3C algorithm, with its rapid convergence and higher long-term rewards, translates directly to more efficient resource allocation in dynamic aerial environments. This becomes particularly significant in urban settings where UAVs must frequently adjust their positions and communication patterns, enabling faster response to topology changes and varying network demands.

The comparative analysis of learning rates provides crucial guidance for system optimization. While rates of 0.005 and 0.015 demonstrate better stability and convergence in our tests, the optimal choice depends heavily on the deployment scenario. Emergency response situations may benefit from higher learning rates to enable rapid adaptation, while long-term monitoring applications like agricultural surveillance might prefer lower rates for more stable performance.

The observed loss function behavior across different configurations reveals important considerations for system reliability. The periodic fluctuations at lower learning rates, rather than being problematic, demonstrate the system’s adaptability to changing conditions—a crucial feature in dynamic UAV networks. The more stable convergence pattern at a learning rate of 0.005 suggests this as an optimal starting point for most deployments, with adjustments based on specific operational requirements.

In summary, these findings suggest that system architects should carefully consider the trade-offs between network size, processing speed, and stability when designing UAV networks. We recommend starting with a learning rate of 0.005 for balanced performance, adjusting upward for scenarios requiring rapid adaptation and downward for those prioritizing stability. Implementation of dynamic parameter adjustment mechanisms can help optimize performance across different operational conditions, particularly in mixed-use scenarios where UAV networks must serve multiple purposes with varying requirements.

## 7. Conclusions

This study introduces an adaptive sharding strategy tailored for drone networks, employing an enhanced version of the A3C algorithm to manage the dynamic and complex characteristics of these environments. Notably, our current algorithm operates within a centralized framework, which, while effective in controlled scenarios, limits its scalability and increases susceptibility to single-point failures in large-scale drone networks. Through extensive simulations, we demonstrated that our approach significantly accelerates data processing speeds and bolsters system security, thereby addressing limitations identified in previous studies. This specialized blockchain sharding system represents a promising solution to enhance the overall performance and reliability of UAV networks, paving the way for more efficient and secure operations in the future. For future enhancements, we propose transitioning to a distributed multi-agent reinforcement learning methodology to achieve genuine decentralized autonomous decision-making, thus further strengthening the system’s resilience and scalability.

## Data Availability

The raw data supporting the conclusions of this article will be made available by the authors on request.
